# Label‐Free Morphometric Profiling of Renal Cell Carcinoma Using Digital Holographic Microscopy and Machine Learning

**DOI:** 10.1002/jbio.70379

**Published:** 2026-11-11

**Authors:** Eva R. Schwark, Yazan A. Almahdi, Ayah A. Farhat, Besa Xhabija

**Affiliations:** ^1^ Department of Natural Sciences University of Michigan‐Dearborn Dearborn Michigan USA

**Keywords:** cell line characterization, digital holographic microscopy, dimensionality reduction, label‐free imaging, machine learning, morphometric profiling, phenotypic biomarkers, quantitative phase imaging, random forest classification, renal cell carcinoma

## Abstract

Renal cell carcinoma (RCC) has a substantial mortality rate among urologic cancers today, and our current knowledge of the disease includes few distinct biomarkers, making early diagnosis particularly difficult. In this study, we utilized digital holographic microscopy (DHM) and quantitative phase imaging (QPI) to investigate trends in cell morphology and optical properties across 38 morphological and optical features in renal carcinoma cell lines UO‐31 and SN12C, and renal epithelial cell line HK2. Dimensionality reduction techniques including PCA, t‐SNE, and Spectral Embedding Analysis enabled clearer differentiation between cancerous and normal cells. Our findings reveal distinct morphological profiles, with UO‐31 and SN12C characterized by greater elongation, coarser features, and lower optical thickness compared to HK2. A Random Forest ensemble classifier achieved a cross‐validated accuracy of 99.50% and AUC values exceeding 0.999 for all three cell lines, with Irregularity and Texture Clustershade emerging as the most influential discriminatory features.

## Graphical abstract

Label‐free digital holographic microscopy and quantitative phase imaging distinguish normal renal epithelial cells from two renal cell carcinoma lines with 99.50% classification accuracy using 38 morphometric features, identifying Irregularity and Texture Clustershade as key biophysical biomarkers of malignancy.

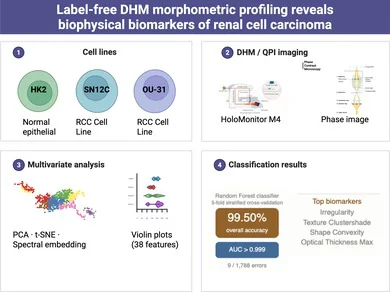

## 1 Introduction

Renal cell carcinoma (RCC) is the most common form of kidney cancer, accounting for over 90% of adult kidney malignancies [1, 2]. RCC predominantly originates from proximal tubule epithelial cells, with its most common subtypes, clear cell and papillary RCC, both arising from proximal tubule epithelium, and is characterized by several heterogeneous subtypes with unique molecular and histopathological characteristics [2, 3]. In 2024, kidney cancer ranked 15th in incidence and 16th in mortality globally, accounting for 442 570  cases and 155 953 deaths  [4, 5]. Despite advances in diagnostic technologies, RCC cases continue to rise, driven by a global increase in the prevalence of risk factors including smoking, obesity, and hypertension [6, 7].

Today, over half of all RCC cases are diagnosed incidentally [2]. In many instances, RCC remains asymptomatic during malignancy, and around 30% of patients reach metastasis by the time of diagnosis [8]. Metastasis to lungs, bone, and liver is most common in RCC cases [9]. Although the survival rate of localized RCC is high (~93%), undetected malignant tumors have a tendency for unpredictable metastasis, contributing to a high mortality rate for metastatic RCC with a 5‐year prognosis of approximately 15%–18% [6, 10]. Due to the heterogeneity of structural and behavioral characteristics, the mechanisms of tumor progression, malignancy, and known biomarkers remain poorly understood [2, 11]. Identifying key RCC biomarkers will advance our understanding of renal cancer, aiding in early tumor detection and treatment [11].

Biological hallmarks of cancer are extremely difficult to identify with qualitative data alone, particularly in structurally diverse cell types such as RCC [12, 13]. Recent advances in digital microscopy have enabled researchers to gather high‐resolution, quantitative data on live cells [14]. Digital Holographic Microscopy (DHM) is a non‐invasive, label‐free, quantitative phase imaging (QPI) technique that can be used to profile morphological and optical characteristics of cell types [15, 16]. Unlike traditional staining methods of cell imaging, DHM captures morphological properties of live cells [17]. High‐resolution QPI reveals extensive morphological data, including cell area, volume, and roughness [16]. Additionally, QPI allows for detailed analysis of cell behavior, including proliferation and motility patterns, which aid in highly detailed cell phenotyping and biomarker identification [15, 16, 17].

The clinical value of a label‐free, quantitative approach becomes clear when set against the diagnostic realities of RCC. Because more than half of RCC cases are found incidentally and roughly a third have already metastasized by the time of diagnosis [2, 8], there is a real need for objective, quantitative tools that can characterize renal cells without the confounding effects of exogenous labels. DHM and QPI are well suited to this need. Since quantitative phase information can resolve nanoscale differences in the morphology and optical density of unstained cells [18, 19], thus a label‐free morphometric signature could, in principle, complement existing cytological and histological workflows. A particularly promising translational route is the label‐free profiling of circulating tumor cells in liquid biopsy, where quantitative phase imaging combined with machine learning has been shown to identify and classify tumor cells directly in flow, avoiding both the invasiveness of solid‐tissue biopsy and the labeling and enrichment artifacts of conventional cytometric assays [20, 21].

Beyond detection, the subjectivity and imperfect reproducibility of histopathological grading remain persistent obstacles to accurate patient stratification in RCC [22, 23]. Consistent with this, phase‐based contrast imaging has been shown to distinguish renal cell carcinoma from normal kidney tissue and to reveal subtype‐associated architecture [24], while label‐free holotomographic imaging combined with machine learning has resolved cancer‐cell states such as drug‐treated versus untreated tumor cells [25]. Because DHM images live cells non‐invasively and over time, it is well positioned for disease and treatment monitoring: it can track dynamic phenotypes such as proliferation and motility [17], and label‐free quantitative phase features combined with machine learning have been used to distinguish drug‐resistant from drug‐sensitive cancer cells [26], all without the destructive endpoint assays that such measurements would otherwise require. We therefore regard the present cell‐line study as a proof of concept: a demonstration that label‐free DHM and machine learning can resolve distinct renal cell phenotypes with high accuracy, establishing a foundation that will need to be validated in patient‐derived cells before it can contribute to earlier detection, stratification, or monitoring in the clinic [27].

Dimensionality reduction techniques improve interpretability of large, high‐dimensional datasets by preserving natural structure and minimizing redundant or irrelevant data [28, 29]. Principal Component Analysis (PCA) is an adaptive, linear method that transforms correlated variables into uncorrelated principal components, revealing global morphometric relationships [28]. T‐Distributed Stochastic Neighbor Embedding analysis (t‐SNE) is a nonlinear method that preserves local relationships, though somewhat at the expense of global relationships, resolving distinct clusters that may uncover biological relationships invisible to PCA [30]. Similarly, Spectral Embedding Analysis constructs a neighborhood graph via local relationships and computing a graph Laplacian [31]. Eigenvectors reveal smooth morphological transitions between clusters, also preserving some global structure [32]. In combination, these dimensionality reduction techniques may reveal a variety of global and local trends with valuable insight into highly specific morphological differences between RCC and normal renal cells.

This study aims to compare morphological and optical properties of two RCC cell lines, SN12C and UO‐31, and one non‐cancerous renal epithelial cell line, HK2 (Figure 1). High‐resolution, non‐invasive DHM and QPI resolve 38 morphological and optical features for each cell line. Multivariate computational analysis identifies a range of trends via dimensionality reduction, encompassing linear, non‐linear, local, and global relationships between data. Additional techniques, including hierarchical clustering, feature importance ranking, radar plot visualization, and predictive classification using a Random Forest ensemble classifier, identify notable trends between cancer and non‐cancer cell types and thoroughly profile each cell line. By identifying features with high discriminatory power, we sought to advance the field of phenotypic profiling using a novel approach. Profiling biomarkers unique to renal cell carcinoma may support future diagnostic strategies and improve methods of treatment and early detection.

**FIGURE 1:**
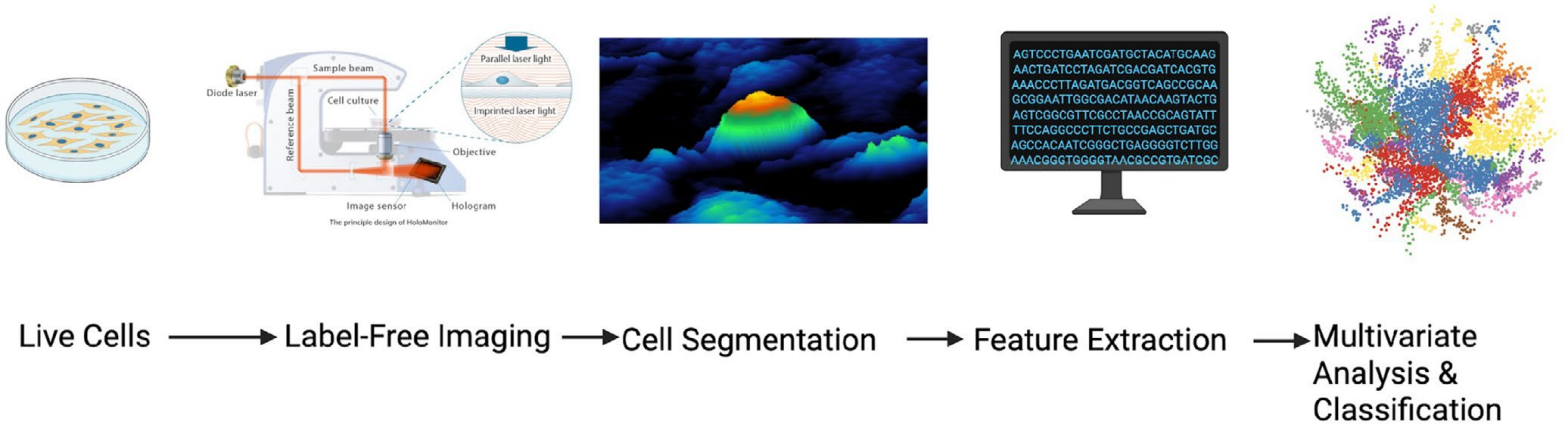
Overview of the label‐free morphometric profiling pipeline. Live renal cells are imaged using Digital Holographic Microscopy (DHM), a label‐free quantitative phase imaging technique. Individual cells are segmented from holographic images, and 38 morphological and optical features are extracted per cell. Extracted features are subjected to multivariate statistical analysis and machine learning‐based classification to differentiate renal cell lines.

## 2 Materials and Methods

### 2.1 Cell Culture, Sample Preparation and 2D and Pseudo‐3D Holographic Image Analysis

All three cell lines, HK2, SN12C and UO‐31 were seeded at 4000 cells per well in the 24‐well tissue‐culture plates (Fisher Scientific). Cells were cultured in RPMI‐1640 medium supplemented with 10% fetal bovine serum (Sigma Aldrich) and 1% penicillin–streptomycin (Fisher Scientific). Cells were incubated at 37°C in a humidified atmosphere with 5% CO_2_. For holographic imaging, the standard plate lids were replaced with 24‐well 14 mm HoloLids (Phase Holographic Imaging, Lund, Sweden; catalog number 71132). Label‐free, live‐cell imaging was conducted using the HoloMonitor M4 holographic imaging microscope (Phase Holographic Imaging, Lund, Sweden). The HoloMonitor M4 uses a 635‐nm diode laser for illumination and a 20X/0.40 objective. HoloMonitor App Suite software, version 4.0.2.752, was used for image acquisition and quantitative phase image generation. Images were acquired at 15‐min intervals over a 48‐h period, with each interval comprising 30 frames per well. Morphological and optical data were subsequently extracted from the captured frames using the Holomonitor App Suite Cell Imaging software(Phase Holographic Imaging, Lund, Sweden), which facilitated Guided End‐Point Assays, Guided Kinetic Assays, and In‐Depth Assays, providing comprehensive quantitative profiles of each renal cell line across the observation period.

Wide‐field holographic images of each renal cell line were acquired continuously throughout the 48‐h observation period (Figure 2A). To improve visual differentiation between cellular and extracellular regions, the image color palette was adjusted within the Coloring panel of the App Suite software from a monochromatic display to a false‐ color scheme in which color reflects optical thickness with the surrounding medium shown in black. Individual cells from each line were further examined through the In‐Depth Analysis: Single Cell Tracking package, which enabled conversion of 2D holographic images into pseudo‐3D reconstructions via the Viewer Options panel. A “Sea” color palette was applied to these reconstructions to enhance contrast across cellular features, with color gradients reflecting variations in cell thickness (Figure 2B).

**FIGURE 2:**
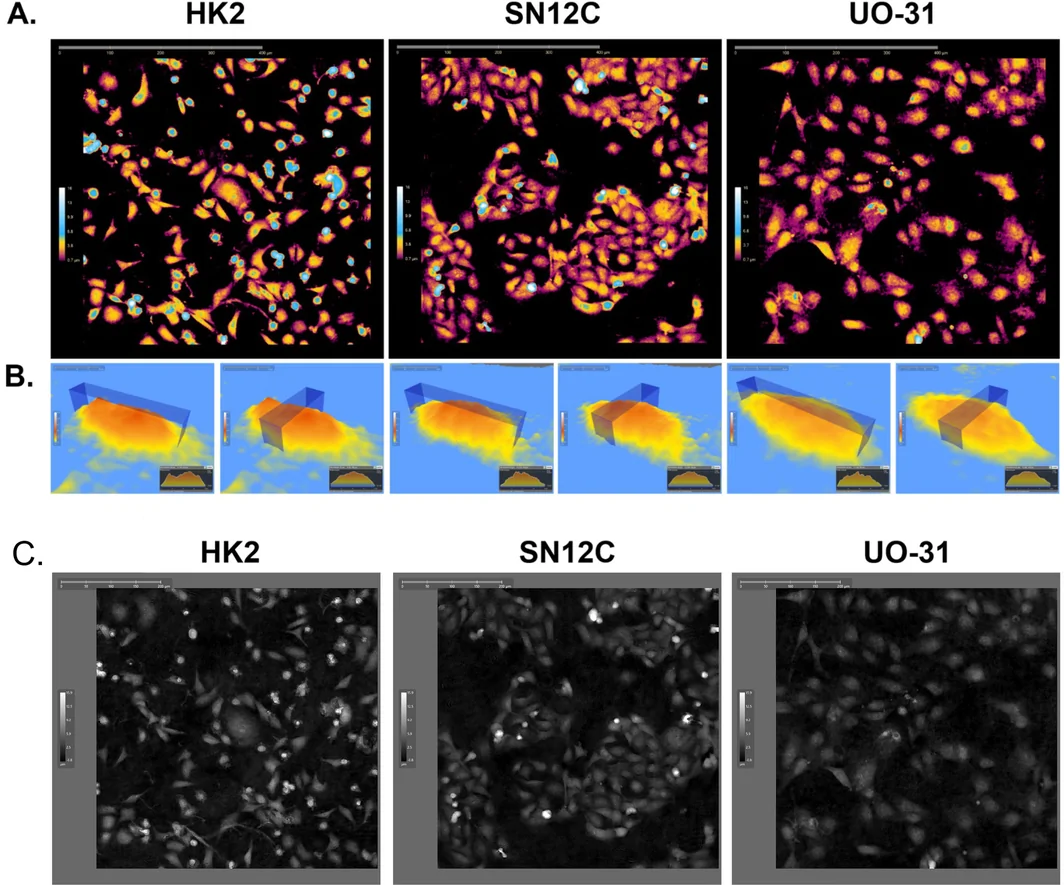
Holographic imaging of renal cell lines reveals morphological differences between normal and cancerous cells. (A) Representative wide‐field holographic images of HK2 (normal), SN12C (cancer), and UO‐31 (cancer) cell lines, displayed using a false ‐color palette in which color reflects optical thickness, ranging from dark purple (thinnest) through orange and yellow to light blue (thickest) with the surrounding median shown in black. Scale bars are shown in each panel. (B) Pseudo‐3D reconstructions of representative individual cells from each line, rendered using a “Sea” color palette to enhance contrast across cellular features; color gradients reflect variations in cell thickness. (C) Representative 2D grayscale quantitative phase images of HK2, SN12C and UO‐31 cells.

### 2.2 Data Preprocessing, Standardization, and Dimensionality Reduction

The extracted morphometric dataset underwent quality control prior to analysis, with cells bearing extreme values beyond three standard deviations from the mean excluded to ensure data integrity. Remaining data were *z*‐score normalized across all 38 features to account for differences in scale and units, and cell observations were labeled by type for all downstream analyses. Three dimensionality reduction techniques were applied to explore the multivariate structure of the data from distinct mathematical perspectives. PCA was performed using the scikit‐learn PCA class, with the first two principal components capturing 43.11% and 24.35% of total variance respectively, revealing global morphological relationships between cell lines. t‐SNE was implemented with perplexity = 40, learning_rate = 200, and *n*_iter = 1000, resolving local cluster structure and fine‐grained separation between the three cell populations. Spectral Embedding was performed with *n*_neighbors = 30 and affinity = ‘nearest_neighbors’, providing additional insight into the manifold geometry of the data. Across all three methods, HK2, SN12C, and UO‐31 cells were represented in green, blue, and red respectively, with plots generated at 600 DPI using Matplotlib and seaborn (Figure 3).

**FIGURE 3:**
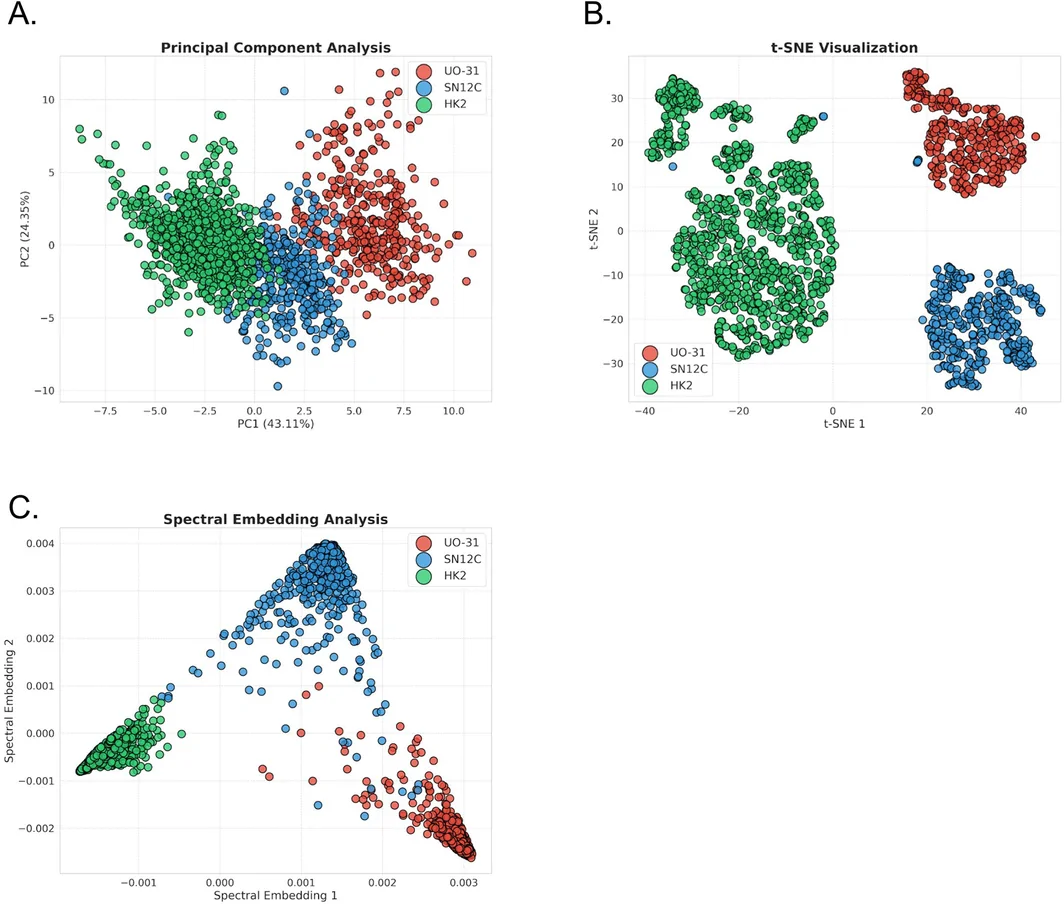
Dimensionality reduction analyses reveal distinct morphological profiles across renal cell lines. (A) Principal Component Analysis (PCA) plot of the 38 morphometric features, with PC1 and PC2 capturing 43.11% and 24.35% of total variance, respectively. (B) t‐SNE visualization (perplexity = 40, learning rate = 200, *n*_iter = 1000) resolving local cluster structure among the three cell lines. (C) Spectral Embedding Analysis (*n*_neighbors = 30) illustrating manifold geometry and morphological transitions between cell populations. In all panels, HK2, SN12C, and UO‐31 cells are represented in green, blue, and red, respectively.

### 2.3 Statistical Comparison of Morphological and Optical Features

Morphological and optical differences between the three cell lines were assessed using one‐way ANOVA for each of the 38 features, followed by Tukey's post hoc test for pairwise comparisons; (Table S1). *p*‐Values were additionally adjusted for multiple comparisons using the Benjamini‐Hochberg false discovery rate procedure. The *p*‐values displayed on each violin plot reflect raw one‐way ANOVA values; Benjamini‐Hochberg adjusted values are provided in Table S1. Feature distributions across HK2, SN12C, and UO‐31 were visualized as violin plots—HK2 in green, SN12C in blue, and UO‐31 in red—with morphological and optical features presented in Figures 4 and 5 respectively, rendered at 600 DPI using Matplotlib and seaborn.

**FIGURE 4:**
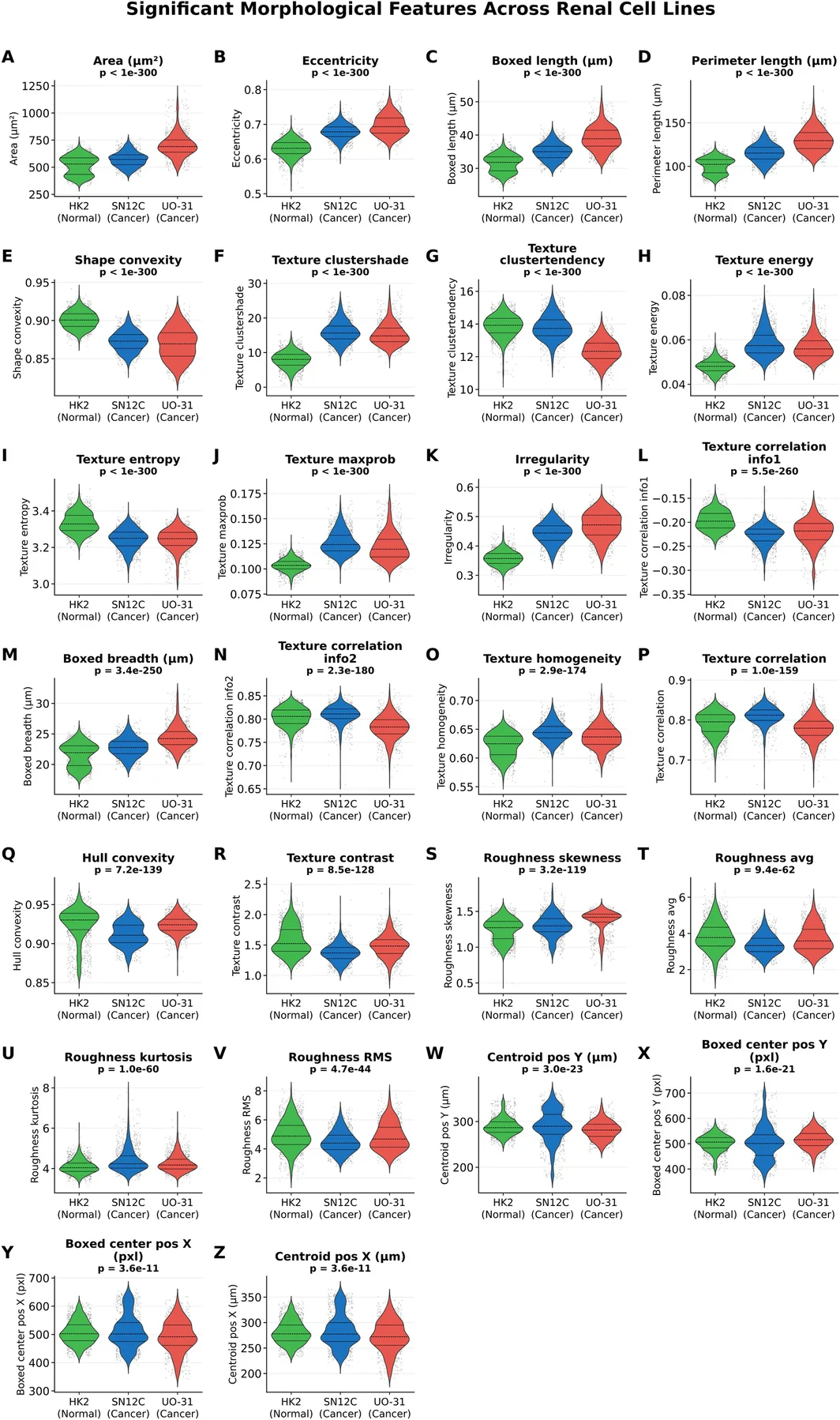
Violin plots of significant morphological features across renal cell lines. Distribution of 26 statistically significant morphological features across HK2 (Normal, green), SN12C (Cancer, blue), and UO‐31 (Cancer, red) cell lines: (A) Area (μm^2^), (B) Eccentricity, (C) Boxed length (μm), (D) Perimeter length (μm), (E) Shape convexity, (F) Texture clustershade, (G) Texture clustertendency, (H) Texture energy, (I) Texture entropy, (J) Texture maxprob, (K) Irregularity, (L) Texture correlation info1, (M) Boxed breadth (μm), (N) Texture correlation info2, (O) Texture homogeneity, (P) Texture correlation, (Q) Hull convexity, (R) Texture contrast, (S) Roughness skewness, (T) Roughness avg., (U) Roughness kurtosis, (V) Roughness RMS, (W) Centroid pos Y (μm), (X) Boxed center pos X (pxl), (Y) Boxed center pos Y (pxl), (Z) Centroid pos X (μm). *p*‐Values reflect one‐way ANOVA results; dashed lines within violins indicate quartiles.

**FIGURE 5:**
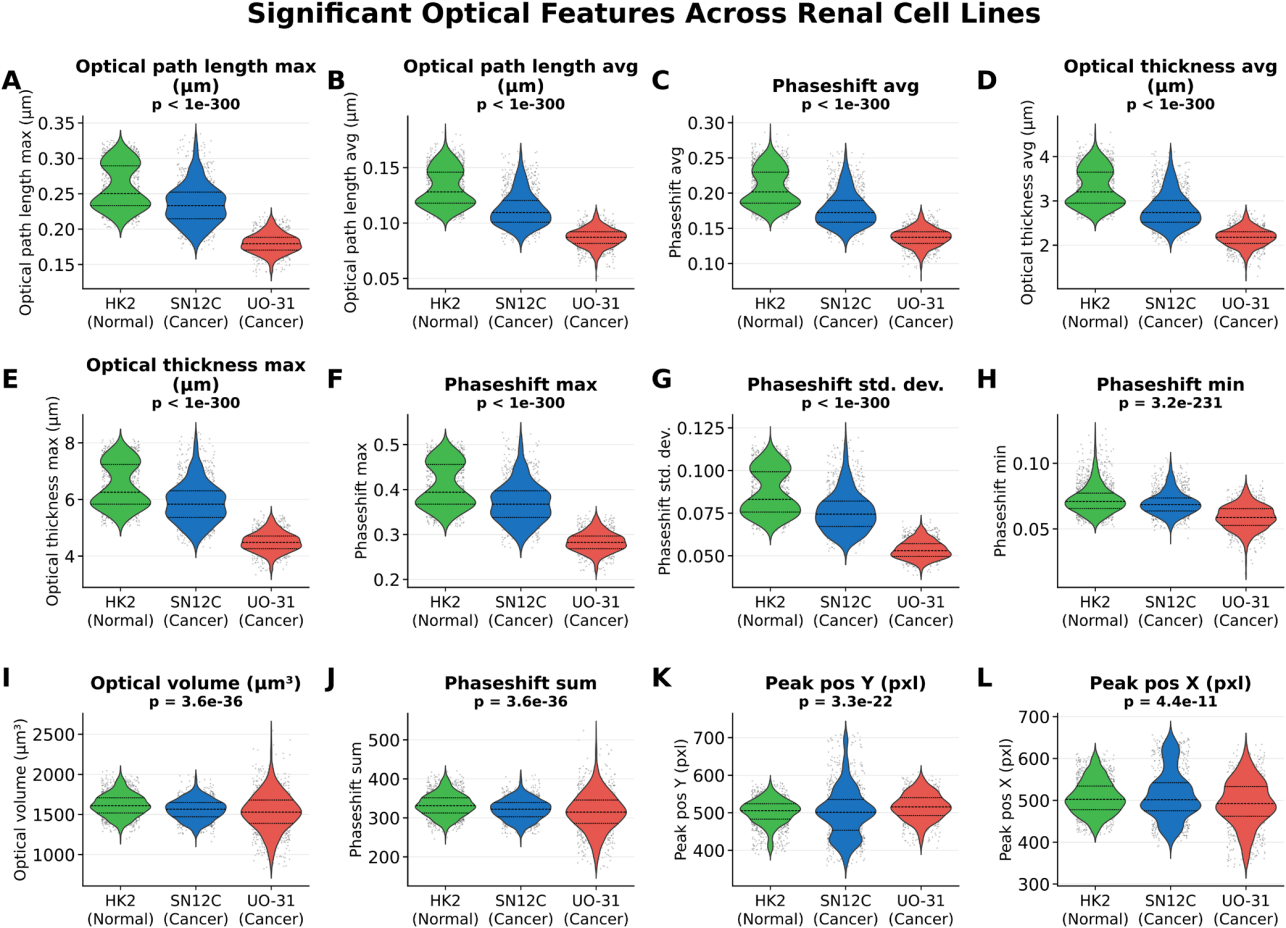
Violin plots of significant optical features across renal cell lines. Distribution of 12 statistically significant optical features across HK2 (Normal, green), SN12C (Cancer, blue), and UO‐31 (Cancer, red) cell lines: (A) Optical path length max (μm), (B) Optical path length avg. (μm), (C) Phaseshift avg., (D) Optical thickness avg. (μm), (E) Optical thickness max (μm), (F) Phaseshift max, (G) Phaseshift std. dev., (H) Phaseshift min, (I) Optical volume (μm^3^), (J) Phaseshift sum, (K) Peak pos Y (pxl), (L) Peak pos X (pxl). *p*‐Values reflect one‐way ANOVA results; dashed lines within violins indicate quartiles.

### 2.4 Hierarchical Clustering, Feature Ranking, and Radar Visualization

To characterize global patterns of morphometric similarity, hierarchical clustering was performed on the 38 *Z*‐scored features across all three cell lines using Euclidean distance and Ward's linkage, with the resulting dendrogram structure presented in the heatmap of Figure 6. Features were additionally ranked by their univariate discriminatory power using the Kruskal–Wallis –log_10_(*p*‐value) and categorized by type—Area, Shape, Optical, Texture, and Other—as shown in Figure 7. This ranking reflects the statistical separation contributed by each feature in isolation and is therefore distinct from the multivariate feature importance reported by the classifier below. A radar plot was further constructed across eight representative features such as Hull convexity, Eccentricity, Area, Perimeter length, Optical thickness avg., Texture contrast, Texture entropy, and Shape convexity, to provide an integrated visual summary of the morphological profiles of the three renal cell lines (Figure 8).

**FIGURE 6:**
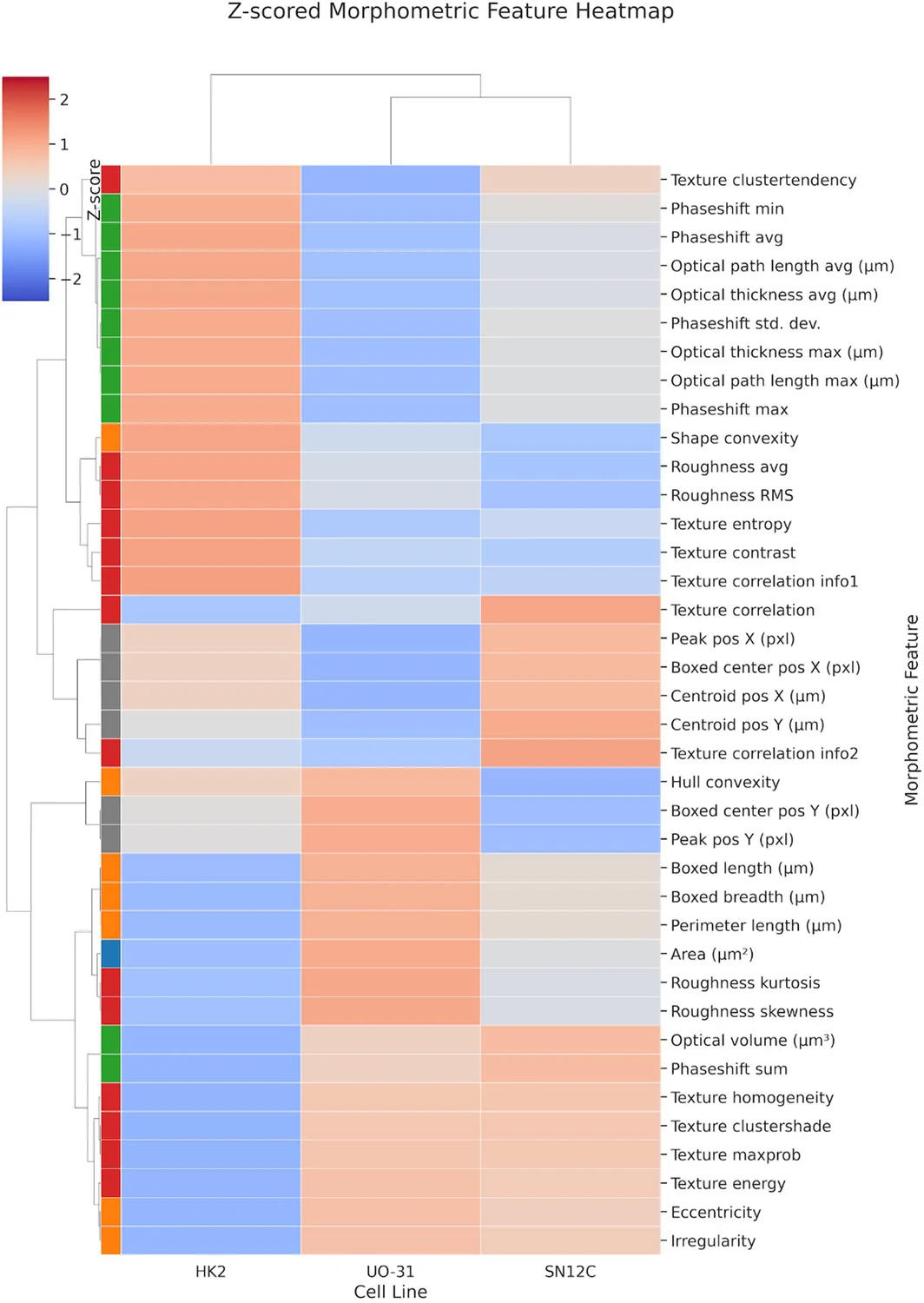
*Z*‐scored morphometric feature heatmap with hierarchical clustering. Heatmap displaying *Z*‐scored values of 38 morphometric features across HK2, UO‐31, and SN12C cell lines. Color scale ranges from blue (low *Z*‐score) to red (high *Z*‐score). Hierarchical clustering was performed using Euclidean distance and Ward's linkage; dendrograms on the left and top group features and cell lines by morphometric similarity, respectively.

**FIGURE 7:**
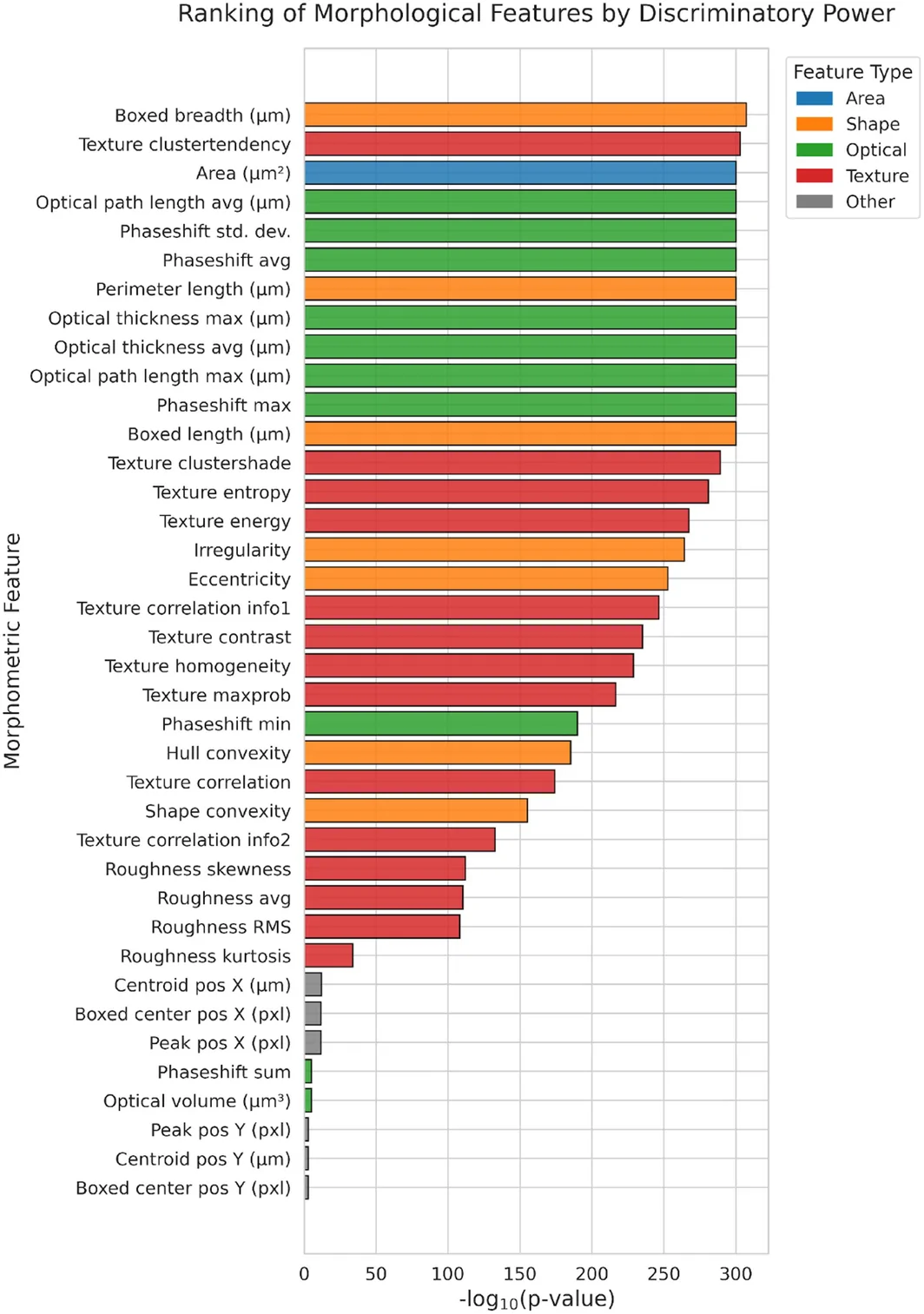
Ranking of morphometric features by univariate discriminatory power. Horizontal bar chart ranking 38 morphometric features by their –log_10_(*p*‐value) derived Kruskal–Wallis test, reflecting the statistical separation contributed by each feature in isolation. Features are color‐coded by type: Area (blue), Shape (orange), Optical (green), Texture (red), and Other (gray).

**FIGURE 8:**
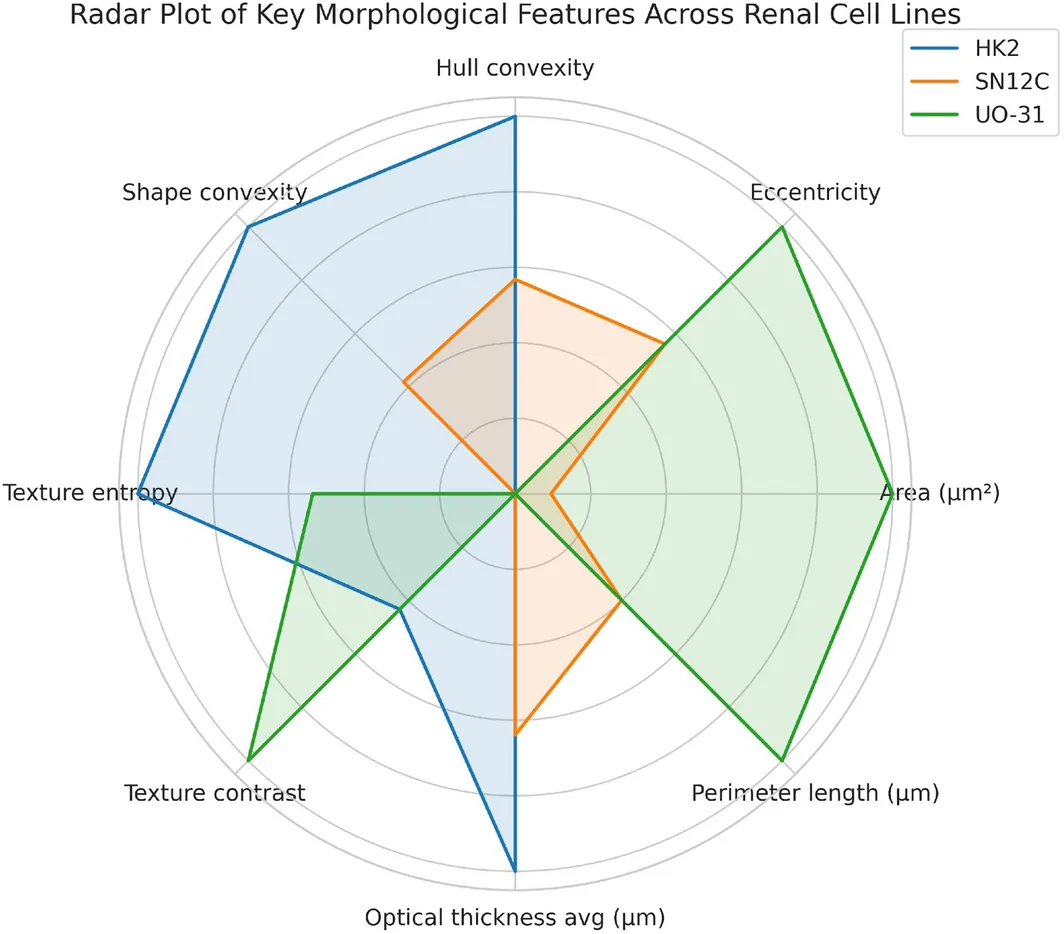
Radar plot of key morphological features across renal cell lines. Normalized radar plot visualizing eight representative morphometric features—Hull convexity, Eccentricity, Area (μm^2^), Perimeter length (μm), Optical thickness avg. (μm), Texture contrast, Texture entropy, and Shape convexity—for HK2 (blue), SN12C (orange), and UO‐31 (green) cell lines, providing an integrated summary of the distinct morphological profiles of each line.

### 2.5 Predictive Classification

A Random Forest [33] ensemble classifier (*n*_estimators = 100, max_depth = None, random_state = 42) was trained on the 32 non‐spatial standardized morphometric features to evaluate the separability of the three renal cell lines. Classification performance was evaluated using stratified fivefold cross‐validation (StratifiedKFold, *n*_splits = 5, shuffle = True, random_state = 42), with predictions aggregated across all folds to produce a single confusion matrix representing all 1788 cells. One‐versus‐rest ROC curves and corresponding AUC values were computed from the cross‐validated predicted probabilities. Feature importance was derived from the mean decrease in Gini impurity across all five trained forests, with standard deviation reported across folds (Figure 9). To further evaluate the specific contribution of the DHM‐derived optical features, we repeated the classification using three feature sets: the full set of 32 non‐spatial features, a morphology‐only set excluding all optical features (22 features), and an optical‐only set (8 features, obtained after collapsing three mutually collinear phase‐derived averages to a single representative). All three models used identical Random Forest settings and the same stratified 5‐fold cross‐validation, and their performance is compared in Table 1. All analyses were implemented in Python using scikit‐learn, Matplotlib, seaborn, SciPy, NumPy, and pandas.

**FIGURE 9:**
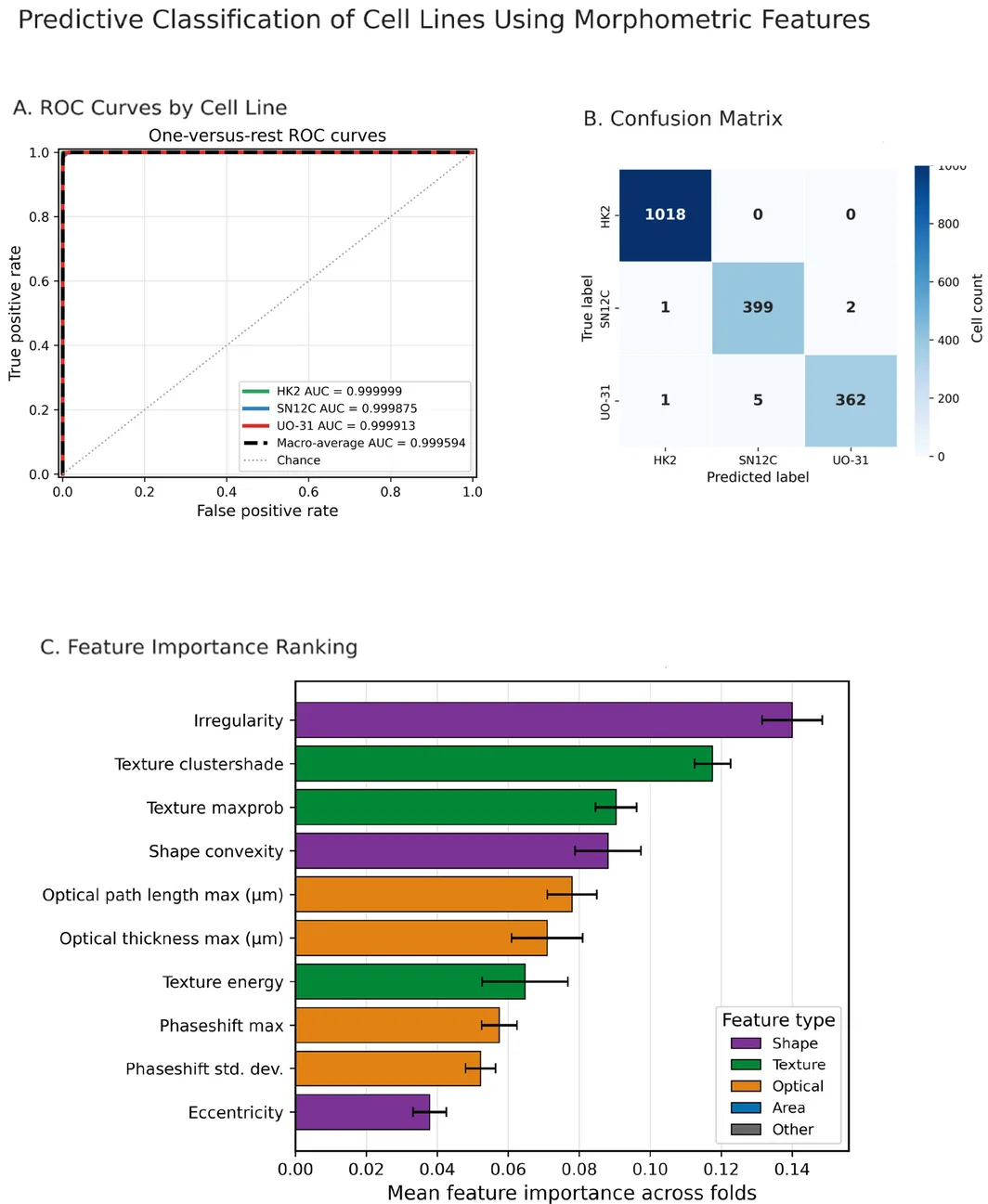
Predictive classification of renal cell lines using morphometric features. (A) One‐versus‐rest ROC curves for HK2 (AUC = 0.999999), SN12C (AUC = 0.999875), and UO‐31 (AUC = 0.999913), with a macro‐average AUC of 0.999594, derived from 5‐fold stratified cross‐validation of a Random Forest ensemble classifier trained on 32 non‐spatial morphometric features. (B) Aggregated confusion matrix across all five folds for 1788 cells, with rows indicating true labels and columns indicating predicted labels. (C) Feature importance ranking based on mean decrease in Gini impurity averaged across all five trained forests; error bars represent standard deviation across folds. Features are color‐coded by type: Shape (purple), Texture (green), Optical (orange), Area (dark green), and Other (gray).

**TABLE 1:** Random Forest classification performance using full, morphology‐only, and optical‐only feature sets.

Feature set	Features (*n*)	CV accuracy	Macro AUC	AUC HK2	AUC SN12C	AUC UO‐31	Misclassified (*n*)	SN12C–UO‐31 errors
Full	32	0.995	0.9999	1	0.9999	0.9999	9	7
Morphology‐only	22	0.9911	0.9995	1	0.9993	0.9993	16	14
Optical‐only	8	0.9804	0.9977	0.9983	0.9969	0.998	35	6

*Note:* Fivefold stratified cross‐validation on 1788 cells (*n*_estimators = 100, random_state = 42). Full = all 32 non‐spatial features; Morphology‐only = 22 non‐optical features; Optical‐only = quantitative‐phase features with three collinear averages reduced to one (8 features). Collapsing these two redundant averages in the full model as well leaves performance essentially unchanged (99.6% vs. 99.5%), confirming the result does not depend on this choice. AUC = area under the one‐vs‐rest ROC curve. Removing optical features nearly doubles the total errors (9 → 16) and the SN12C–UO‐31 confusions (7 → 14); optical features alone classify at 98.04%. *Source:* rf_ablation on ‘renal data.xlsx’, 2026‐07.

## 3 Results

### 3.1 Pseudo‐3D Microscopy and Single‐Cell Images Reveal Key Differences Between RCC and Normal Renal Cells

In Figure 2, pseudo‐3D microscopy images contrast the larger, more elongated RCC cell lines with the notably more uniform and smaller HK2. Representative 2D grayscale quantitative phase images of the three cell lines are shown in Figure 2C. Panel A presents representative wide‐field holographic images of each cell line, while Panel B displays pseudo‐3D reconstructions of individual cells, together illustrating differences in cell morphology and optical depth across the three renal lines. The images reveal that HK2 is more uniform than both UO‐31 and SN12C. RCC cell lines appear more elongated with greater irregularity. It may be noted that UO‐31 is considerably larger in terms of area and length than SN12C, and most distinctly contrasts patterns unique to HK2.

### 3.2 Dimensionality Reduction Analysis Reveals Distinct Properties Between and Among Normal and Cancerous Kidney Cells

In order to measure the variance in morphological features between the normal HK2 cells and cancerous UO‐31 and SN12C cells, the morphological data was analyzed with the dimensionality reduction techniques PCA, t‐SNE, and Spectral Embedding Analysis. Each offered unique insights into the morphological profiles of the HK2, UO‐31, and SN12C cells. In Figure 3A, the PCA plot reveals largely distinct characteristics between the cell lines. By capturing the greatest variance along both axes, PCA reveals global patterns in the data. Each cell line occupies a largely distinct region in the PCA space, suggesting significant morphological differences between the normal and cancerous cells, though it may be noted that some overlap is observed between SN12C and UO‐31 along the PC1 axis, indicating a degree of shared morphological characteristics among the cancerous lines. It also indicates that cells within their own lines are more likely to be similar than to cells from other lines. Furthermore, the multiple UO‐31 subclusters and great UO‐31 and HK2 dispersion suggest some degree of heterogeneity within the cell lines themselves. The t‐SNE plot validates the findings in the PCA analysis in Figure 3B, mapping high‐dimensional patterns into clusters while preserving the local pattern structure. It is apparent that the three cell lines occupy different regions in the t‐SNE 1 and t‐SNE 2 axes, suggesting distinct morphological profiles. The internal clusters within all three cell lines suggest a degree of heterogeneity within the normal and cancerous cell lines as well, thus suggesting variation between and among the cell lines. However, the prominent separation and distinct internal clusters of the HK2 cells suggest a greater degree of variance in the noncancerous cellular morphology compared to the cancerous. Spectral Embedding Analysis provides further insights into the inherent morphological patterns by preserving the manifold structure, as seen in Figure 3C. It may be observed that there are prominent clusters at each vertex, suggesting the majority of the cells exhibited characteristics similar to their cell line. Data points along the edges and towards the inside, away from the vertices, however, suggest more mixed profiles, less characteristic of their line's “archetype.” It may be observed that there is more dispersion among the cancerous cell lines than the noncancerous line, corroborating with the PCA findings that there is a degree of similarity in the cancerous morphological profiles. Thus, the Spectral Embedding Analysis suggests that the noncancerous cells are dissimilar from the cancerous cells, which share similar, yet distinct, morphological profiles among themselves.

### 3.3 Violin Plots Reveal Distinct Morphological and Optical Profiles, but Similar Positional Trends Between the RCC Lines

Violin plots visualize the distribution of cell data across the renal cell lines, revealing distinct morphological profiles in Figure 4; full one‐way ANOVA and Tukey pairwise statistics for all features are reported in Table S1 With respect to the parameters Area, Eccentricity, Boxed Length, Boxed Breadth, and Perimeter Length (Figure 4A–D,M), the cancerous UO‐31 and SN12C cell lines generally displayed greater median values than the noncancerous HK2 cells. Between the cancerous cells, UO‐31 consistently had greater values. These observations suggest that the cancerous renal cell lines are more elongated with greater areas and perimeters, though UO‐31 exhibits this trend most prominently. Furthermore, the data is most concentrated around the median for UO‐31 and SN12C compared to HK2, which has a more varied distribution; thus, the findings suggest greater similarity between cancerous cells and their own cell line compared to noncancerous cells.

This pattern is not observed with the Shape Convexity parameter, however (Figure 4E). The HK2 data is concentrated at higher values compared to both of the cancerous lines, suggesting that the noncancerous cells tend to have more regularly outlined cell boundaries than their cancerous counterparts. Regarding the cancerous cells, the SN12C and UO‐31 cells have relatively similar median values; however, UO‐31 has a broader distribution. These observations suggest that although there is a similar central tendency for both cancer lines, there is more variation and heterogeneity in convexity among the UO‐31 cells.

Furthermore, the texture parameters highlighted in the violin plots reveal distinct patterns among the normal cells and RCC cells, including the Texture Clustershade, Clustertendency, Energy, Entropy, and Max Prob (Figure 4F–J). Across the Clustershade and Energy parameters, it may be observed that both of the RCC cell lines have similar medians and distributions, which are higher than the HK2 cells. On the other hand, HK2 cells exhibit higher medians in the Clustertendency and Entropy features. These patterns suggest that the cancerous cells are dominated with coarse, repeated features that display asymmetry; on the other hand, the normal cells have more balanced texture patterns with higher complexity. Additionally, the distinct profiles across the Irregularity parameter provide greater insight into the differentiation of each renal line (Figure 4K). Regarding Irregularity, the UO‐31 cells are characterized by the greatest median and distribution, with HK2 cells bearing the lowest median and compact distribution. Thus, it is suggested that the UO‐31 cancer cells vary the most from the circumference of a circle, with the noncancerous cells being the most circular among the renal lines observed.

There is similar variability in distribution across the Texture Homogeneity, Correlation, Correlation Info2, and Contrast parameters (Figure 4N–R), as each cell line may be characterized by broad distributions and/or significant tail regions. Additionally, the HK2 cells are characterized by greater Hull Convexity values, suggesting the noncancerous cells are more characteristic of a perfect convex shape with fewer variations in cell thickness (Figure 4Q). The understanding of these distinct profiles is notable upon examination of the Roughness parameters, including Roughness Skewness, Average, Kurtosis, and RMS (Figure 4S–V). Roughness Average and RMS are characterized by similar trends, with HK2 cells bearing greater medians and broader distributions compared to the cancerous cell lines; among the cancerous lines, UO‐31 had broader distributions. These findings suggest that the HK2 cells may be characterized by rougher surfaces on average compared to the cancerous cells. On the contrary, the UO‐31 cancerous cells had values concentrated at higher values compared to the other cell lines for the Roughness Skewness property, suggesting a texture with more prominent peaks than indents. SN12C and HK2 were characterized by lower median values, though HK2 had a more prominent tail. With respect to Roughness Kurtosis, or the sharpness of the surface height distribution, HK2 cells displayed lower median values than both cancerous lines, suggesting lower extremity in surface height features among the noncancerous cells. The UO‐31 and SN12C lines were characterized by higher, more concentrated kurtosis values, indicating less uniform surface feature sharpness relative to HK2.

The violin plots reveal similarities among the renal cell lines when tested against the geometric and positional parameters. With respect to the Centroid Position X and Y features (Figure 4W,Z), all three renal lines are characterized by similar median values, suggesting similar locations of the center of mass within the cells. However, the SN12C cells bore broader distributions, suggesting greater heterogeneity within this cell line. These patterns may also be observed in the Boxed Center Position X and Y parameters (Figure 4X–Y), revealing the coordinates of the geometric center. All three renal lines exhibited similar medians, though SN12C had a far broader distribution, with pronounced tails, compared to the more concentrated HK2 and UO‐31 values. This suggests that while the three cell lines tend to have similar geometric center positions, there is far more variability in the SN12C cell line.

### 3.4 Optical Features Reveal Distinct Patterns Among Renal Cell Lines

The optical features of the renal cell lines are highlighted in violin plots (Figure 5). Across the majority of the optical parameters tested, the noncancerous HK2 cell line displayed the greatest optical path length values, followed by the SN12C cancer cell line, and the UO‐31 cell line had the lowest values. Specifically, the HK2 cell line displayed greater Optical Path Length and Thickness median values compared to the cancerous renal cell lines, followed by the SN12C line (Figure 5A,B,D,E). This suggests that the noncancerous cell line is thicker with a greater optical path length compared to the cancerous lines; meanwhile, the HK2 and SN12C lines had more varied distributions compared to the UO‐31 line, suggesting less varied Optical Path Length and Thickness in UO‐31 cells. A similar pattern is observed among the Phaseshift Avg, Max, Std. Dev, and Min parameters, in which the HK2 noncancerous cells have greater median values and distribution while the UO‐31 cells are characterized by the lowest values concentrated around the median (Figure 5C,F–H). This reveals that the HK2 cells had greater phase shifts measured within the cell areas, suggesting inherent differences in the structure of the noncancerous and cancerous cell lines. Overall, throughout these parameters, the UO‐31 line consistently displayed lower median values and more compact distributions than the HK2 and SN12C cell distributions.

However, the Optical Volume and Phaseshift Sum values reveal similar medians across all three renal cell lines, with more distributed UO‐31 values and more compact HK2 and SN12C values (Figure 5I,J). This suggests that although the three cell lines have similar optical volume and phaseshift sum values, the UO‐31 cell line has more variation in measurements. Furthermore, the three renal cell lines have similar Peak Position *X* and *Y* medians and overlapping values, with SN12C cells bearing greater distributions (Figure 5K,L). This suggests that the SN12C cells have a more diverse distribution of locations of the thickest cell region.

### 3.5 *Z*‐Scored Morphometric Heatmap Highlights Feature Trends Differentiating RCC and Normal Renal Cells

Hierarchical clustering of cell lines as well as morphometric features clearly groups similar patterns in morphology between cell types. In Figure 6, 38 *Z*‐scored morphometric features are displayed across all three cell lines. Features with similar *Z*‐scores are distinctly clustered by the dendrogram on the left, while cell lines with similar characteristics are clustered by the dendrogram on the top. Clustering patterns suggest clear morphometric differences between all three cell lines, with HK2 being the most distinct from UO‐31 and SN12C. HK2 shows notably higher *Z*‐scores for the top feature cluster, including Texture Entropy, Contrast, and Correlation, Roughness Avg and RMS, Max Optical Path Length and Thickness, Avg Optical Path Length and Thickness, and Phaseshift Min, Max, and Avg. In contrast, SN12C and UO‐31 display lower‐than‐average *Z*‐scored values for the same cluster. Additionally, HK2 shows lower‐than‐average *Z*‐scores for the bottom feature cluster, including Irregularity, Eccentricity, Optical Volume, Area, Perimeter Length, Boxed Breadth, and Boxed Length. Consistent with the violin plot findings, SN12C and UO‐31 are scored significantly higher than average for this same feature cluster, reflecting their greater size, elongation, and irregularity relative to HK2. Similar to findings in Figure 4, UO‐31 has consistently higher *Z*‐scored values than SN12C, highlighting the stark contrast between HK2 and UO‐31. Furthermore, the strong co‐clustering of SN12C and UO‐31 in the top dendrogram highlights their shared cancerous morphological profile relative to HK2, suggesting that morphological markers may distinguish cancerous and normal renal cells.

### 3.6 Ranked Feature Importance Plot Reveals Discriminatory Power Across Morphological and Optical Features

The Ranked Feature Importance Plot was used to uncover which cellular property in isolation is useful in differentiating between the renal cell lines (Figure 7). The plot reveals that Boxed Breadth has the greatest discriminatory power, followed closely by Texture Clustertendency, Area, and optical properties. The plot thus elucidates that area, shape, and optical properties are most significant in differentiating the renal cell lines. On the other hand, positional properties, such as Centroid and Boxed Center positions, were ranked the least significant in differentiating the cells.

### 3.7 Radar Plot Compares Key Morphological and Structural Features Defining the RCC and Normal Cell Profiles

The Radar Plot visualizes key features that define the three renal cell lines HK2, SN12C, and UO‐31, helping compare physical and textural characteristics (Figure 8). In the plot, it is observed that the HK2 cell line displays high values in the Hull Convexity, Shape Convexity, Texture Entropy, and Optical Thickness Avg features. The UO‐31 dominates in the remaining features, including Eccentricity, Area, Perimeter Length, and Texture Contrast. The SN12C line, on the other hand, has moderate values across most of the features. These observations summarize key findings about each cell line. That is, the HK2 cells are thicker with more convex shapes and complex textures compared to UO‐31 cells, while the SN12C cells show more intermediate morphology when compared to the other two cell lines.

### 3.8 Predictive Classification Metrics Support Robust Morphometric Feature Contrast Between Cell Types

Figure 9 quantitatively confirms the findings of the unsupervised analyses above. A Random Forest ensemble classifier trained on 32 non‐spatial morphometric features demonstrated robust discriminative power across all three renal cell lines under 5‐fold stratified cross‐validation. As demonstrated in Figure 9A, one‐versus‐rest ROC curves for HK2, SN12C, and UO‐31 achieved AUC values of 0.999999, 0.999875, and 0.999913, respectively, with a macro‐average AUC of 0.999594, indicating near‐perfect separability of the three cell lines based on morphometric features alone. The aggregated confusion matrix across all five folds (Figure 9B) presents the classification performance across all 1788 cells. Of these, only 9 were misclassified, yielding an overall cross‐validated accuracy of 99.50%. It may be noted that the majority of misclassifications occurred between the two cancerous cell lines, 5 UO‐31 cells predicted as SN12C and 2 SN12C cells predicted as UO‐31, consistent with the partial overlap observed between SN12C and UO‐31 in the dimensionality reduction analyses (Figure 3). HK2 cells were classified with perfect accuracy across all folds, reflecting the distinct morphological separation of the noncancerous line from both RCC lines.

Figure 9C presents the feature importance ranking derived from the Random Forest model, reflecting the mean decrease in Gini impurity averaged across all five trained forests, with error bars representing standard deviation across folds. Irregularity and Texture Clustershade ranked as the most influential features, with mean importances of 0.1400 ± 0.0085 and 0.1176 ± 0.0051, respectively. Among the top 10 features, texture and optical features proved most influential in the multivariate classification context, despite shape and area features such as Boxed Breadth and Area appearing most distinctive in the univariate discriminatory power analysis of Figure 7. This contrast highlights the complementary value of both univariate and multivariate approaches in characterizing morphometric differences between the renal cell lines.

In order to assess how much of this discriminatory power derives specifically from the quantitative phase information, we compared classifiers trained with and without the optical features (Table 1). Removing the optical features left the separation of the noncancerous HK2 line essentially intact, yet nearly doubled the overall misclassifications, from 9 to 16, with the additional errors concentrated between the two morphologically similar RCC lines, with misclassifications between SN12C and UO‐31 rising from 7 to 14. The optical features alone, only eight in number, still classified the three lines at 98.04% accuracy. Our data indicates that the quantitative phase features carry substantial and largely independent discriminatory information, sharpening the more difficult malignant–malignant boundary rather than the normal–cancer one.

## 4 Discussion

This study revealed distinct morphological and optical characteristics of SN12C, UO‐31, and HK2. In order to visually represent differences between normal and cancerous renal cell lines, pseudo‐3D images of each cell line were reconstructed using QPI. By comparing morphometrics of each cell line, holographic images revealed potential distinctions between RCC and non‐cancer cell lines, pertaining to features such as length, width, thickness, and structural heterogeneity. In Figure 2, RCC cell lines appeared more elongated and optically thinner, while HK2 appeared uniform and smaller, with greater structural heterogeneity within its cell line. Structural variation, namely a greater average Boxed Breadth, is suggestive of phenotypic trends in the HK2 cell line. Cultured proximal tubule epithelial cells are known to form dome‐like structures [34], which may contribute to the notably greater optical thickness, phase shift values, and Boxed Breadth observed in HK2 relative to the RCC lines in the quantitative analyses below.

Dimensionality reduction techniques further highlight the distinct morphologies between the noncancerous cells and RCC by uncovering salient patterns [28, 30] (Figure 3). The PCA plot clearly separates the UO‐31, SN12C, and HK2 cell lines, suggesting distinct morphological features. It also reveals a degree of heterogeneity within the cell lines themselves, due to the presence of distinct subclusters, and a degree of similarity between the cancerous UO‐31 and SN12C cells from overlap. Similarly, the t‐SNE plot highlights distinct morphological profiles, indicating a clear separation between each cell line yet suggesting internal variation prominently within the noncancerous HK2 cells. The Spectral Embedding Analysis aligns these findings with distinct clusters; it also corroborates with the PCA findings, suggesting a degree of similarity between the UO‐31 and SN12C cells due to overlap.

The analysis of dimensionality reduction establishes that the three renal cell lines occupy largely distinct regions of morphometric space, yet these methods are unsupervised and inherently qualitative. They reveal that a separation exists, but they neither quantify it nor identify the features that drive it, and they offer no means of classifying new, unlabeled cells. This is what motivated the supervised Random Forest analysis. The classifier complements the embeddings in several respects: it quantifies separability through cross‐validated performance metrics, ranks features by their multivariate discriminatory contribution, yields a model that generalizes to previously unseen cells, and rigorously tests the hardest discrimination, that between the two morphologically similar RCC lines, which visual inspection of the embeddings alone cannot confirm. The unsupervised and supervised analyses are therefore complementary. One discovers structure, whereas the other validates and quantifies it.

Violin plots of each morphological, textural, geometric, and optical feature analyzed expanded on the overarching patterns identified through microscopy and dimensionality reduction (Figures 4 and 5). It is suggested that the RCC cells, overall, were more elongated with greater perimeters and areas, with UO‐31 bearing the greatest values. UO‐31 also prominently exhibited the greatest variance from the circumference of a circle and more prominent surface peaks. HK2, on the other hand, had more regularly outlined cell boundaries, a more balanced texture, and a more uniform shape with fewer changes in cell thickness. While SN12C was moderate in the majority of the properties measured, it was characteristically most heterogeneous in the geometric parameters of Centroid Position and Boxed Center Position. Thus, while the three cell lines displayed similar coordinates for the center of mass and geometric center, SN12C had broader distributions, suggesting more variability in their geometry. These findings were further visualized in a radar plot providing a concise representation of the key morphological profiles of each cell line (Figure 8).

Analysis of optical features revealed the HK2 cells as optically thicker with greater optical path lengths and phase shifts compared to both RCC lines (Figure 5). It is also suggested that HK2, SN12C, and UO‐31 exhibit overlap in the location of the thickest cell region, though there is suggested to be greater variability in SN12C. Additionally, multivariate feature representation revealed key relationships and insights within the complex datasets. The *Z*‐scored morphometric heatmap (Figure 6) showed a clear visual separation between the two RCC cell lines and HK2, with texture features including Texture Entropy, Contrast, Correlation, and Roughness significantly elevated in HK2 compared to SN12C and UO‐31. Meanwhile, shape characteristics including Irregularity, Eccentricity, Boxed Length, and Boxed Breadth were higher in RCC cell lines than HK2, providing an insightful view into the trends which separate cancerous and noncancerous cell lines as seen in Figures 4 and 5. Among the morphometric features analyzed, the Ranked Feature Importance Plot (Figure 7) reveals that Boxed Breadth is the most significant feature in discriminating between the cell lines in isolation, while positional properties were the least important. The biological significance of Boxed Breadth as a distinguishing feature between HK2 and the RCC lines warrants further investigation, particularly given the dome‐forming propensity of proximal tubule epithelial cells noted above. Future studies may elucidate the broader functional significance of this finding.

Furthermore, a Random Forest ensemble classifier evaluated under 5‐fold stratified cross‐validation demonstrated near‐perfect discriminative power across all three cell lines, with one‐versus‐rest AUC values of 0.999999, 0.999875, and 0.999913 for HK2, SN12C, and UO‐31 respectively [33] (Figure 9A). Of the 1788 cells evaluated across all folds, only 9 were misclassified, yielding an overall cross‐validated accuracy of 99.50%. It may be noted that the majority of misclassifications occurred between the two RCC cell lines‐ 7 of the 9 errors involved SN12C and UO‐31‐ consistent with the partial morphological overlap observed between these lines in the dimensionality reduction analyses (Figure 3). HK2 cells were classified with perfect accuracy across all folds, further underscoring the distinct morphological profile of the noncancerous line. The ablation analysis (Table 1) further clarifies where this discriminatory power originates: morphological features alone separate normal from cancerous cells almost perfectly, whereas the optical, quantitative‐phase features are what resolve the two RCC lines from one another. The two feature families are therefore complementary, and both contribute to the near‐perfect separation of the three cell lines. Overall, the model robustly supports the conclusion that morphometric features derived from DHM may successfully distinguish between normal and cancerous renal cell lines.

Taken together with the conclusions drawn from Figures 2, 3, 4, 5, 6, 7, 8, particularly the violin plots (Figures 4 and 5), *Z*‐Scored Morphometric Feature Heatmap (Figure 6), and Ranked Feature Importance Plot (Figure 7), a set of key features characteristic of RCC and normal renal cells may be inferred. Figure 6 depicts a stark contrast between HK2 and the RCC cells across many characteristics shared with Figure 9C. Normal renal cells were notably higher than average for optical features including Optical Path Length Max, Optical Thickness Max, and Phaseshift Max—three of the 10 most important features in Figure 9C. In Figure 6, Shape Convexity was notably elevated in HK2 while Irregularity and Texture Clustershade were greater in the RCC lines—all three features correspondingly ranked among the most important in Figure 9C. Standout characteristics noted across the analyses, including Boxed Breadth, Texture Clustertendency, Irregularity, and Texture Clustershade, warrant further investigation as potential major morphometric biomarkers of RCC and normal renal epithelial phenotype.

One further point of interest pertains to the contrasting structural heterogeneity of normal and cancerous renal cells. Cluster overlap in Figure 3 and rare misclassifications of RCC cell lines suggest a degree of intercellular similarity between SN12C and UO‐31. We note, too, that the study already incorporates two RCC lines whose morphological profiles are closely related. SN12C and UO‐31 overlap in the PCA and Spectral Embedding projections and account for the majority of the classifier's errors (7 of the 9), yet they remain separable at 99.50% accuracy. The methodology is thus already tested on a pair of morphologically similar populations, and its success on this difficult discrimination is one of the central findings of the study. Previous studies have addressed tumor heterogeneity in clear cell RCC and its potential to make tumor evaluation and treatment difficult [35, 36]. Additionally, pathologic grading systems for RCC are imperfect, yet grading may influence prognosis in early‐stage tumors [22, 23]. While morphology has been the classic indicator of tumor grade, heterogeneity as well as the lack of comprehensive study of the wide range of RCC subtypes contributes to grading difficulties and the absence of a definitive method. Uncovering nuanced details about RCC subtypes may dispel some issues in treatment, helping to improve patient outcomes and develop more selective therapies.

Several limitations of the present study warrant acknowledgement. The analyses were conducted on established cell lines rather than primary patient‐derived samples, which may not fully recapitulate the morphological heterogeneity observed in vivo. Furthermore, only two RCC subtypes are represented, and the generalizability of these findings to other RCC variants remains to be established. It may also be noted that certain positional features, including Centroid Position and Boxed Center Position, that showed statistical significance may partially reflect imaging field position rather than intrinsic biological differences, and their inclusion as discriminatory features should be interpreted with appropriate caution. Additionally, correlation analysis revealed that several optical features, specifically Phaseshift avg., Optical thickness avg., and Optical path length avg., are mathematically equivalent up to constant scaling factors, reflecting their derivation from the same underlying phase measurement. These features should not be treated as fully independent predictors, and future studies should consider dimensionality reduction or feature selection strategies to address this redundancy prior to classification. In order to test the methodology across a wider range of morphological phenotypes, it would be interesting to further investigate a broader panel of renal cell lines in future studies, in particular additional RCC subtypes and primary, patient‐derived samples that more closely recapitulate the heterogeneity seen in vivo. These closely related populations would place an even more demanding test on the approach, and we need a better understanding of how these morphometric signatures behave across the full diversity of RCC before they can be translated toward clinical use.

DHM, QPI, and machine learning allow for the identification of morphometric biomarkers characteristic of multiple RCC variants and normal renal cell types, with features such as Irregularity, Texture Clustershade, Optical Path Length, and Optical Thickness emerging as particularly promising candidates. The label‐free, non‐invasive nature of DHM preserves an ideal cell environment, accurately capturing cell characteristics in high resolution without the confounding effects of exogenous labels [15]. By collecting high‐resolution morphometric data on renal cell lines using DHM, performing thorough statistical analysis, and validating findings using a supervised machine learning classifier, this study presents a novel approach in phenotypic profiling that may contribute to improvements in diagnostic accuracy, treatment stratification, and patient prognosis in renal cell carcinoma.

## Funding

This research was funded by the College of Arts, Sciences and Letters Internal Research Fund, University of Michigan‐Dearborn.

## Conflicts of Interest

The authors declare no conflicts of interest.

## Supporting information


**Table S1:** One‐way ANOVA and Tukey post hoc comparisons, and Benjamini‐Hochberg adjusted *p*‐values for morphometric features across HK2, SN12C, and UO‐31 cells.

## Data Availability

The data supporting the findings of this study are available from the corresponding author upon reasonable request.
